# Membrane-bound receptor for advanced glycation end products (RAGE) is a stable biomarker of low-quality sperm

**DOI:** 10.1093/hropen/hoae064

**Published:** 2024-11-07

**Authors:** Jill Browning, Magda Ghanim, William Jagoe, Jennifer Cullinane, Louise E Glover, Mary Wingfield, Vincent P Kelly

**Affiliations:** School of Biochemistry & Immunology, Trinity College Dublin, Dublin, Ireland; School of Biochemistry & Immunology, Trinity College Dublin, Dublin, Ireland; School of Biochemistry & Immunology, Trinity College Dublin, Dublin, Ireland; Merrion Fertility Clinic, Dublin, Ireland; Merrion Fertility Clinic, Dublin, Ireland; School of Medicine, University College Dublin, Dublin, Ireland; School of Medicine, Trinity College Dublin, Dublin, Ireland; Merrion Fertility Clinic, Dublin, Ireland; School of Medicine, University College Dublin, Dublin, Ireland; School of Medicine, Trinity College Dublin, Dublin, Ireland; School of Biochemistry & Immunology, Trinity College Dublin, Dublin, Ireland

**Keywords:** RAGE, male infertility, infertility, biomarker, sperm biochemistry, sperm quality, semen analysis

## Abstract

**STUDY QUESTION:**

Does receptor for advanced glycation end products (RAGE) on the surface membrane of the sperm cell function as a biomarker of low-quality sperm?

**SUMMARY ANSWER:**

Membrane-bound RAGE at a cellular level directly correlates with low sperm motility, high cell permeability, decreased mitochondrial function, DNA fragmentation, and higher levels of apoptosis.

**WHAT IS KNOWN ALREADY:**

RAGE has previously been measured by ELISA in low-quality sperm in diabetic men and has been shown to correlate with DNA fragmentation (terminal deoxynucleotidyl transferase dUTP nick end labelling (TUNEL) assay).

**STUDY DESIGN, SIZE, DURATION:**

Semen samples were recovered from 60 non-obese, non-diabetic and non-smoking subjects, washed with fresh media, and analysed directly or purified further by differential gradient centrifugation (DGC) or fractionated by direct swim-up before being analysed for sperm motility and molecular health parameters, including cell membrane permeability, cell death, mitochondrial membrane potential, DNA fragmentation, and RAGE protein expression.

**PARTICIPANTS/MATERIALS, SETTING, METHODS:**

Sperm motility assessments were carried out by computer-assisted sperm analysis (CASA) on 1000 spermatozoa for washed samples and 300 spermatozoa for purified samples. Molecular sperm health parameters were evaluated using flow cytometry with the use of the following markers: DAPI for cell membrane permeability, Annexin V/DAPI for cell death (apoptosis and necrosis), MitoTracker^®^ Red CMXRos for mitochondrial membrane potential, TUNEL assay for DNA fragmentation and 8-hydroxy-2-deoxyguanosine for identification of oxidative damage to sperm DNA, and contrasted to membrane-bound RAGE expression levels, which were evaluated using an anti-RAGE monoclonal mouse antibody.

**MAIN RESULTS AND THE ROLE OF CHANCE:**

RAGE protein was shown to be present on the acrosomal and equatorial regions of sperm, with the levels of membrane bound receptor strongly correlating with poor sperm health across all parameters tested; motility (*R*^2^ = 0.5441, *P* < 0.0001) and mitochondrial membrane potential (*R*^2^ = 0.6181, *P* < 0.0001) being of particular note. The analysis was performed at a single cell level thereby removing confounding complications from soluble forms of the RAGE protein that can be found in seminal plasma. The expression of the RAGE protein was shown to be stable over time and its levels are therefore not subject to variation in sample handling or preparation time.

**LARGE SCALE DATA:**

N/A.

**LIMITATIONS, REASONS FOR CAUTION:**

Inclusion criteria for this study were non-diabetic, non-obese and non-smoking participants to assess the distribution of RAGE expression in the general population, thereby excluding disease conditions that may increase RAGE expression in sperm or contribute to low sperm quality. The study does not address how RAGE expression may be affected in other patient subpopulations or disease states associated with male infertility. Sperm analysis by flow cytometry is not amenable to the study of males with a low sperm count.

**WIDER IMPLICATIONS OF THE FINDINGS:**

Results of this study suggest that RAGE expression is a molecular maker of sperm cell health, which may be used for improvements in assisted reproduction through the removal of RAGE expressing sperm and facilitate in the diagnoses of unexplained infertility through its use as a biomarker of male infertility.

**STUDY FUNDING/COMPETING INTEREST(S):**

The study was funded by the Irish Research Council under the Government of Ireland Programme (GOIPG/2015/3729) and the Enterprise Ireland Innovation Partnership Programme (IP-2020-0952). All authors declare no competing interests.

WHAT DOES THIS MEAN FOR PATIENTS? Sperm issues are identified in 50% of infertility cases; however, in spite of this, infertility research has focused mostly on females. This study tests the potential of using the receptor for advanced glycation end products (RAGE) protein, which is present on the outer membrane of sperm cells, as a biomarker of sperm health.

In this study, we compared the levels of RAGE protein to a number of other measures of sperm health typically used in fertility clinics. The data revealed that increased RAGE protein levels is associated with poor sperm health, as confirmed by multiple different tests. The protein levels are stable over time and are not affected by sample preparation or handling, indicating that it is more specific compared to other existing sperm health assessments.

Further studies are necessary to confirm whether the RAGE protein could be used as a biomarker of male infertility and help improve the processes of assisted reproduction.

## Introduction

Infertility, sometimes also referred to as subfertility, is formally defined as a failure to conceive after 12 months of regular, unprotected sexual intercourse (Vander Borght and Wyns, 2018). It is also considered to be a disease which generates disability as an impairment of function, according to the WHO (World Health Organisation) (Zegers-Hochschild *et al.*, 2017). Estimates of infertility vary depending on the region of the world, but it is assumed that it affects between 8% and 12% of couples of reproductive age globally (Vander Borght and Wyns, 2018). In approximately 20–30% of cases, the sole cause is deemed to be a male or sperm factor; however, sperm issues are identified in 50% of overall infertility cases (Vander Borght and Wyns, 2018). Moreover, a significant decline in sperm count and sperm quality parameters has been observed globally in recent years, particularly in developed countries, giving rise to concerns about the negative impact of the modern lifestyle on male fertility (Nassan *et al.*, 2018; Salas-Huetos *et al.*, 2019; Fonseca *et al.*, 2022; Levine *et al.*, 2023; De Jonge *et al.*, 2024). Despite this, infertility research has tended to be primarily focused on females.

Male infertility can arise for a variety of underlying reasons, including varicocele, genetic alterations, systemic diseases, and altered semen parameters (Oehninger *et al.*, 2000). However, the single greatest contributory factor to male infertility is sperm damage due to oxidative stress and resultant DNA fragmentation, estimated to account for between 30% and 80% of cases (Showell *et al.*, 2014; Agarwal *et al.*, 2020). Not only does DNA fragmentation affect sperm quality, it has also been found to negatively impact embryo quality, implantation rates and miscarriage rates as well as the health and wellbeing of the offspring (Agarwal *et al.*, 2020).

Currently, various semen analysis methods are available; however, no consensus has been reached on the reliability or validity of these techniques (Tanga *et al.*, 2021). Traditional semen analysis involves examining the physical and structural characteristics of the seminal fluid and sperm, including volume, pH, liquefaction, concentration (sperm count), morphology, sperm motility and progression, etc. (Boitrelle *et al.*, 2021; World Health Organization, 2021). The nature of these tests raises concerns regarding the possible impact of environmental conditions such as the storage temperature of the sample (Tanga *et al.* 2021). Moreover, although the use of computer-assisted sperm analysis (CASA), flow cytometry, NucleoCounter SP-100, or automated sperm morphometry analysis (ASMA) are becoming more popular to analyse sperm counts, morphology, and/or motility, it is still common that these analyses are performed using phase-contrast microscopy, which means that the results can depend heavily on subjective factors and the experience of the embryologist (Tanga *et al.*, 2021).

Biochemical assays allow testing of the function of the male reproductive tract (prostate, epididymis and seminal vesicles) and focus on the seminal fluids rather than the sperm itself. Levels of proteins, zinc, fructose, and neutral glucosidase may be examined, along with peroxidase positive cells to test for leukocyte content to detect a possible infection (World Health Organization, 2021). Other methods allow for structural examination of sperm including examination of sperm vitality, i.e. tests to differentiate between viable and non-viable sperm. These include dye exclusion staining based on the permeability of their membranes, sperm agglutination with immunoassays for antisperm antibodies, or assessments of chromatin integrity and DNA damage (Vasan, 2011; Tanga *et al.*, 2021; World Health Organization, 2021; Agarwal *et al.*, 2022). During the past two decades a number of tests have been introduced for the analysis of sperm DNA fragmentation. These include terminal deoxynucleotidyl transferase (TdT)-mediated deoxyuridine triphosphate (dUTP)-nick-end labelling (or TUNEL), single cell gel electrophoresis assay (SCGE, also known as the Comet assay), chromomycin A3 (CMA3)-staining, *in situ* nick translation, DNA breakage detection fluorescence in situ hybridization (DBD-FISH), acridine orange (AO) test, sperm chromatin dispersion test (SCD), and the sperm chromatin structure assay (SCSA). These tests are considered to be of higher specificity and accuracy than traditional approaches to semen analysis (Sakkas and Alvarez, 2010). Regrettably, DNA fragmentation tests are not routinely provided due to their high cost and the multistep, non-standardized preparation protocol that may leave room for variability and error (Ribeiro *et al.*, 2017; Tanga *et al.*, 2021).

In addition to being useful for the diagnosis of male factor infertility, extracellular markers that correlate with the degree of sperm DNA damage and the quality of the sperm would be highly valuable for the identification and removal of damaged sperm prior to assisted reproductive technology. Significant efforts have been made over the last three decades to identify a more sophisticated marker. Intracellular markers of DNA fragmentation, oxidative DNA damage, oxidative stress, mitochondrial function, and sperm cell death have been identified (Aitken *et al.*, 1989; Wei and Kao, 2000; Agarwal and Said, 2003; Nakada *et al.*, 2006; Cambi *et al.*, 2013). However, these require invasive internal labelling procedures, leaving the sperm non-viable afterwards.

The receptor for advanced glycation end products (RAGE) is a central signalling molecule of the innate immune system and belongs to a class of cell-surface, pattern recognition receptors (Neeper *et al.*, 1992; Schmidt *et al.*, 1992) encoded at the major histocompatibility complex (MHC) class III locus (Lee and Park 2013). It exists in numerous forms as either a membrane-bound, soluble or secretory protein. RAGE is long established as a key contributor to severe chronic pathologies including neurodegeneration, atherosclerosis, cardiovascular diseases, osteoarthritis, and diabetes (Xie *et al.*, 2013). Under normal conditions, the expression of RAGE is very low (Ramasamy *et al.*, 2009). However, during disease, the production of RAGE is highly induced on the cell surface by a number of different ligands such as advanced glycation end products (AGE), high mobility group box-1 (HMGB1), S100/calgranulins, phosphatidylserine, and C3a (Ramasamy *et al.*, 2009). In addition, RAGE expression is further enhanced after immune activation or infection and many RAGE ligands are secreted by monocytes, macrophages, neutrophils, and leukocytes, which are likely to be crucial for the initiation and propagation of a RAGE-dependent inflammatory response (Ramasamy *et al.*, 2009; Xie *et al.*, 2013).

RAGE has previously been shown to be present in low-quality sperm in diabetic men, where a strong correlation between total RAGE levels and DNA fragmentation (TUNEL assay) was observed (Mallidis *et al.*, 2007; Karimi *et al.*, 2011). To date, only the soluble form of RAGE has been examined in male infertility in the healthy general population (Charalampidou *et al.*, 2017). As the receptor exists in membrane bound and soluble isoforms, there remains an outstanding question of which form correlates with sperm health. Furthermore, it is not clear that the levels of RAGE expression at a single cell level correlate with other sperm health parameters. Thus, this study endeavours to explore these questions in more detail.

In order to avoid the increase in RAGE expression levels due to previously reported factors such as diabetes, we analysed semen samples from non-diabetic, non-obese, and non-smoking men. Exclusively membrane-bound RAGE expression on sperm was tested in this population of men by flow cytometry, and sperm motility was evaluated by CASA. Here, we show that RAGE expression on the sperm membrane correlates with mitochondrial dysfunction, cell death, DNA fragmentation, and low sperm motility. Moreover, we also observed that purifying sperm using conventional techniques, namely DGC and direct swim-up methods, allowed for the removal of RAGE expressing spermatozoa, further indicating the presence of membrane-bound RAGE on low-quality sperm. Membrane-bound RAGE could function as an appropriate biomarker for the identification and negative selection of low-quality sperm for couples experiencing challenges in conceiving.

## Materials and methods

### Ethical approval

This study was carried out using semen samples from non-identifiable subjects attending Merrion Fertility Clinic with signed permission from the donors and ethical approval from the School of Medicine Research Ethics Committee, Trinity College Dublin (20160902). The research was carried out at Trinity Biomedical Sciences Institute, Trinity College Dublin.

### Study population and participants

In total, 60 men attending Merrion Fertility Clinic for a fertility assessment between August 2017 and July 2018 were invited to participate in this project and written informed consent was received from all participants. Samples from participants with type I or type II diabetes, smokers and/or individuals with a BMI >29.9 were excluded.

### Sample collection and analysis

Semen samples were collected after 2–5 days of sexual abstinence. Samples were collected in sterile plastic containers (Sarstedt, Nümbrecht, Germany) and allowed to liquefy for 30 min at 37°C. Semen analysis was performed within 1 h of ejaculation. All samples were subject to conventional analyses for semen volume (ml), sperm concentration (×10^6^/ml), sperm morphology and motility, according to WHO recommendations (World Health Organization, 2010).

### Sperm morphology assessment

Sperm morphology assessments were carried out using the simple normal/abnormal classification. Pre-stained slides (TestSimplets, Waldeck, Münster, Germany) were used following strict Kruger (Tygerberg) criteria. Briefly, a small drop of semen was added to the centre of the coverslip, placed carefully onto the pre-stained area of the slide, and incubated for 15 min at room temperature (RT). A small drop of oil was placed on the centre of the coverslip and the 100× oil immersion lens on an Eclipse E400 microscope (Nikon, Tokyo, Japan) was used to assess morphology. Approximately 200 sperm were assessed and the percentage of normal (score > 4% normal) or abnormal (score of <4% normal) forms were determined.

### Sperm motility assessment

Sperm motility assessments were carried out by CASA using the Hamilton Thorne software (Hamilton Thorne, Beverly, MA, USA) and a Zeiss microscope (Zeiss, Jena, Germany), following WHO and manufacturers’ guidelines. Briefly, semen samples were resuspended using Pasteur pipettes and diluted 1 in 3 in sperm wash media. A volume of 3 µl of diluted semen was added to the CASA microscope slide (Nifa Technologies, Leeuwarden, Netherlands), placed onto the pre-heated stage (37°C) and allowed to settle until no drift was apparent on screen. Motility assessment was evaluated by capturing at least 1000 sperm cell tracks per slide for unprocessed samples and 300 for processed samples. Total cell count (×10^6^/ml), percentage immotile (IM), non-progressively motile (NP) and progressively motile (PR) sperm and average path velocity (VAP) for NP and PM sperm were recorded for each sample.

### Sperm washing

Seminal plasma was removed by washing spermatozoa to 0.5 ml of sperm wash media (Irvine Scientific, Santa Ana, CA, USA) and centrifugation at 1500 × *g* for 1 min at RT, repeated twice. The final sperm pellet was resuspended in 0.1 ml sperm wash media prior to downstream experiments. The sample is hereafter referred to as ‘washed spermatozoa’.

### Sperm preparation

Semen samples were purified by DGC or by direct swim-up. For DGC, the PureSperm 40/80 separation media (NidaCon, Mölndal, Sweden) was used. A volume of 1.5 ml of 40% lower layer separation media was added to a 15 ml falcon tube followed by 1.5 ml of 80% upper layer separation media. A minimum volume of 1 ml of the semen sample was loaded onto the gradient and centrifuged at 400 × *g* for 20 min at RT. Approximately 2.5 ml of the upper layer was gently removed using a sterile Pasteur pipette and the sperm pellet was washed twice with 2 ml sperm wash media and centrifuged at 380 × *g* for 5 min at RT. The washed pellet, containing highly motile purified sperm, was resuspended in 0.5 ml of sperm wash media. For direct swim-up, a minimum 1 ml of seminal plasma was washed twice in 2 ml sperm wash media by centrifugation at 380 × *g* for 5 min at RT. The washed sperm pellet was gently resuspended and layered underneath a 1-ml volume of multipurpose handling media (Irvine Scientific, Santa Ana, CA, USA), pre-equilibrated at 37°C and incubated for 30 min at RT. Approximately 0.8 ml of the top layer, containing highly motile purified sperm, was removed.

### Quantitative assessment of sperm cell health

Measurement of mitochondrial membrane potential (MMP), cell permeability, and cell death were determined using MitoTracker^®^ Red CMXRos (Invitrogen, Waltham, MA, USA), DAPI (Invitrogen), and Annexin V-PeCy7 (Invitrogen), respectively. Per sample, 1 × 10^6^ spermatozoa were washed once with 0.5 ml sperm wash media followed by a two-step wash with 0.5 ml PBS with 1% FBS. MMP was evaluated by incubation with 6.25 nM MitoTracker^®^ Red CMXRos for 15 min at 37°C. Cell permeability was evaluated by incubation with 35 ng/ml DAPI for 5 min at 37°C in 0.1 ml PBS with 1% FBS, protected from light. Samples were washed twice with 0.5 ml of PBS with 1% FBS prior to analysis. Then 1 × 10^6^ sperm were washed into 0.5 ml of 1× Annexin V binding buffer prior to evaluating apoptosis and necrosis using 5 µl of Annexin V-PE-Cy7 in 0.1 ml 1× binding buffer and incubating for 15 min at RT.

### Quantitative assessment of RAGE protein expression

Measurement of RAGE protein expression was determined using an Alexa Fluor 647 conjugated monoclonal mouse anti-human RAGE antibody (Santa Cruz Biotechnology, Dallas, TX, USA). Per sample, 1 × 10^6^ spermatozoa were placed in FACS tubes with 100 µl of PBS with 1% FBS and incubated with 2 µg/ml of RAGE-AF647 antibody for 1 h at 37°C, protected from light. Samples were washed twice with 0.5 ml of PBS with 1% FBS prior to analysis.

### Quantitative assessment of DNA fragmentation

Measurement of DNA fragmentation was determined using the TUNEL assay (Invitrogen), following the manufacturer’s guidelines with minor modifications. After washing in PBS with 1% FBS, 5 × 10^6^ washed sperm cells were fixed with 4% PFA for 30 min and washed twice using PBS with 1% FBS. Fixed cells were permeabilized by incubating in 0.1 ml 0.1% Triton X-100, 0.1% sodium citrate in PBS for 2 min on ice, and washed twice with 0.5 ml of wash buffer, supplied with the kit. Fixed and permeabilized cells were incubated with a TdT TUNEL reaction (TdT enzyme, BrdU, reaction buffer) for 1 h at 37°C, washed twice with 0.5 ml of rinse buffer, and incubated with the mouse anti-BrdU-AF488 antibody for 30 min at RT. Cells were washed twice with 0.5 ml PBS with 1% FBS and counterstained with 10 µl of PI in RNaseA buffer, supplied with the kit. Cells were then analysed by LSR Fortessa Cytometer (BD, Franklin Lakes, NJ, USA). Oxidative DNA damage was measured by assessing the levels of 8-hydroxy-2-deoxyguanosine (8-HOdG) in the samples using an anti-DNA/RNA (15A3) antibody conjugated to FITC (Novus Biologicals, Littleton, CO, USA). After washing in PBS with 1% FBS, 5 × 10^6^ washed sperm cells were fixed with 4% PFA for 30 min and washed twice using PBS with 1% FBS. The cells were then stained with 2 µg/ml of anti-DNA/RNA (15A3)-FITC DNA damage antibody in permeabilization buffer (0.1% sodium citrate, 0.1% Triton X-100 in PBS, pH 7.2) for 1 h at 37°C. Following staining, the samples were washed twice with 500 μl of 1% NGS-PBS solution. Cells were analysed by a LSR Fortessa Cytometer (BD Biosciences, Franklin Lakes, NJ, USA).

### Analysis of sperm cell health using flow cytometry

For flow cytometry data collection, a minimum of 10 000 single events were recorded for each sample at a flow rate of 200 events per second using the LSR Fortessa flow cytometer (BD Biosciences) and analysed by FlowJo™ Software for Mac v10.9.0 (BD Biosciences). DAPI was measured using the 450/50 band pass filter, CMX Ros using the 610/20 filter, PE-Cy7 using the 780/60 filter, AF647 using the 670/14 filter and AF488 and FITC using the 530/30 filter. Spermatozoa were gated using FSC-A and SSC-A in the flame-shaped region to remove debris and cells other than spermatozoa. Doublets were omitted using the FSC-A and FSC-W dot-plot. Each fluorophore was selected and gated against FSC-A with rectangular gates used to select negative and positive populations. A quadrant plot was used to analyse apoptotic and necrotic sperm cells.

### Confocal microscopy analysis of RAGE and mitochondrial visualization

For microscopic evaluation of RAGE and visualization of mitochondria in sperm, slides were viewed using a Leica SP8 Gated STED confocal microscope (Leica, Wetzlar, Germany). For RAGE localization analysis, 1 × 10^6^ washed unprocessed human sperm cells were incubated with 4 µg/ml mouse anti-human RAGE primary antibody (Santa Cruz Biotechnology, Dallas, TX, USA) for 1 h at 37°C, followed by 2 µg/ml goat anti-mouse AF647 secondary antibody (ThermoFisher, Waltham, MA, USA) for 30 min at 37°C. For visualization of mitochondria in sperm, 1 × 10^6^ human sperm cells (purified via DGC) were incubated with 0.5 µM MitoTracker^®^ Red CMXRos for 15 min at 37°C. Stained cells were fixed with 0.5 ml 4% PFA pH 7.2 for 20 min at RT and incubated with 70 ng/ml DAPI. Slides were prepared by adding 20 µl of stained sperm cells onto poly-l-lysine microscope slides and air-drying at 37°C prior to adding mounting medium and a coverslip.

### Statistical analyses

Statistical analyses were performed using GraphPad Prism version 10.0.2 for Mac (GraphPad Software, San Diego, CA, USA). A parametric paired *t*-test was used for motility and RAGE expression comparison as well as for comparing the flow cytometry markers’ expression. All data was assumed to be normally distributed. Values are expressed as ± SD. A *P*-value of <0.05 is considered as statistically significant. The Pearson’s correlation test was performed to analyse the correlation between RAGE protein expression and motility. A one-way ANOVA test, with a two-tailed *P*-value, was used to compare flow cytometry marker expression across unprocessed and processed samples. A *P*-value of <0.05 was considered as statistically significant.

## Results

In this study, we test the hypothesis that the membrane-bound form of RAGE on sperm is a robust indicator of sperm quality. Samples were obtained voluntarily from non-diabetic, non-smoking and non-obese men, aged 37.8 ± 4.7 (mean ± SD) years, who were undergoing fertility treatment.

**Figure 1.**
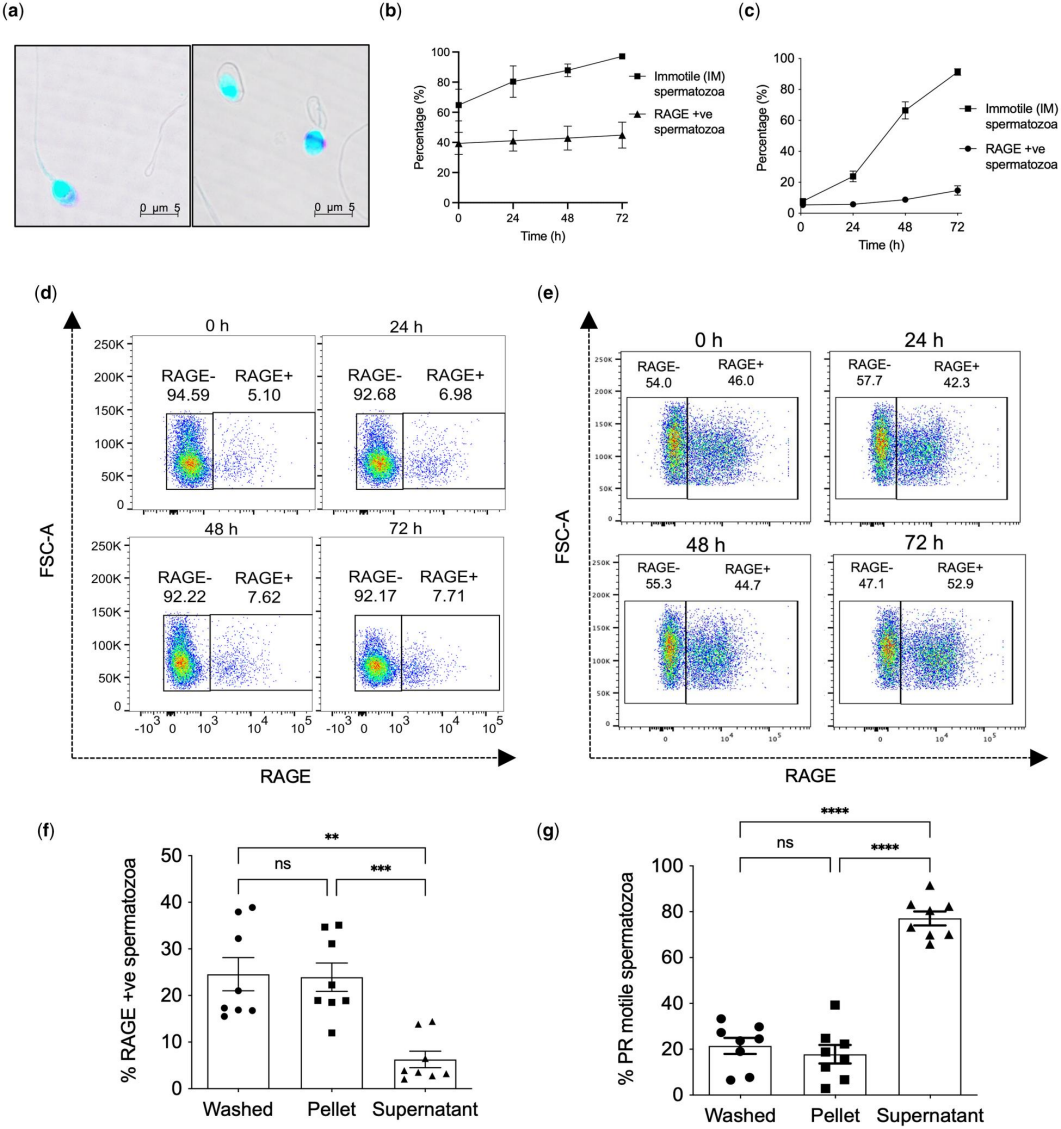
**Membrane-bound RAGE is a stable marker of low-quality sperm**. (**a**) RAGE localization at the acrosome and equatorial band on the sperm cell head as shown by confocal microscopy. (**b** and **c**) Line-graph showing the proportion of IM sperm (analysed using CASA) and sperm expressing RAGE (analysed using flow cytometry) in (**b**) neat (washed) sperm and (**c**) DGC purified spermatozoa when incubated over 72 h, n = 6; % mean ± SEM. (**d** and **e**) Representative flow cytometry data showing RAGE expression in (**d**) DGC purified and (**e**) neat washed spermatozoa over time (0–72 h). (**f** and **g**) Proportion of (**f**) sperm expressing RAGE as measured by flow cytometry and (**g**) PR sperm as measured by CASA in neat washed sperm and the pellet (low-quality) and supernatant (high-quality) fractions of the swim-up purification, n = 15; % mean ± SEM. One-way ANOVA; **P < 0.01, ***P < 0.001, ****P < 0.0001. IM, immotile sperm; CASA, computer-assisted semen analysis; DGC, differential gradient centrifugation; PR, progressively motile sperm.

Using a mouse anti-human RAGE antibody, we confirmed the localization of membrane-bound RAGE at the equatorial mid-piece and acrosomal region of the sperm head, as shown by confocal microscopy (Fig. 1a); corroborating findings of a previous study (Mallidis *et al.*, 2007). Moreover, we demonstrate that the expression of membrane-bound RAGE is stable. Over a 24-h time period, both non-purified (washed) human sperm and sperm purified by DGC showed only a slight increase in RAGE expression levels from 39.4 ± 7.4% to 41.1 ± 6.9% (mean ± SEM) (washed, Fig. 1b) and 5.4 ± 0.9% to 5.8 ± 0.5% (mean ± SEM) (DGC, Fig. 1c), respectively. The stability in the levels of RAGE on the sperm surface was further demonstrated by the modest increases in values at 72 h relative to the 0 h timepoint.

By contrast, marked increases in the proportion of immotile sperm were observed over the same time period. The population of immotile sperm in the unpurified (washed) sample rose from 64.8 ± 10.5% at 0 h to 97.1 ± 1.7% (mean ± SEM) at 72 h, whereas the DGC purified sample showed an increase from 7.7 ± 1.9% at 0 h to 91.3 ± 5.1% (mean ± SEM) at 72 h (Fig. 1b–e). The facile changes that were observed in sperm motility upon exposure to environmental factors, contrast with the expression of RAGE, whose stability following semen production could be inferred to be reflective of the expression status in the individual donor.

To determine whether RAGE expression is associated with low motility and low-quality sperm, a comparative analysis of RAGE expression and progressive motility was performed on the supernatant and pellet fractions obtained after swim-up purification of neat sperm samples. The supernatant fraction containing high-quality sperm showed significantly lower RAGE expression levels as well as significantly higher sperm motility compared to the pellet fraction and unpurified, neat (washed) sample (Fig. 1f and g). The pellet fraction containing low-quality sperm, in turn, showed significantly higher RAGE expression and significantly lower progressive sperm motility than the supernatant fraction. This in turn suggests a close link between membrane RAGE expression and poor sperm health. Moreover, it indicates that the RAGE positive sperm in semen samples can be separated from RAGE negative sperm using conventional methods of sperm purification.

**Figure 2.**
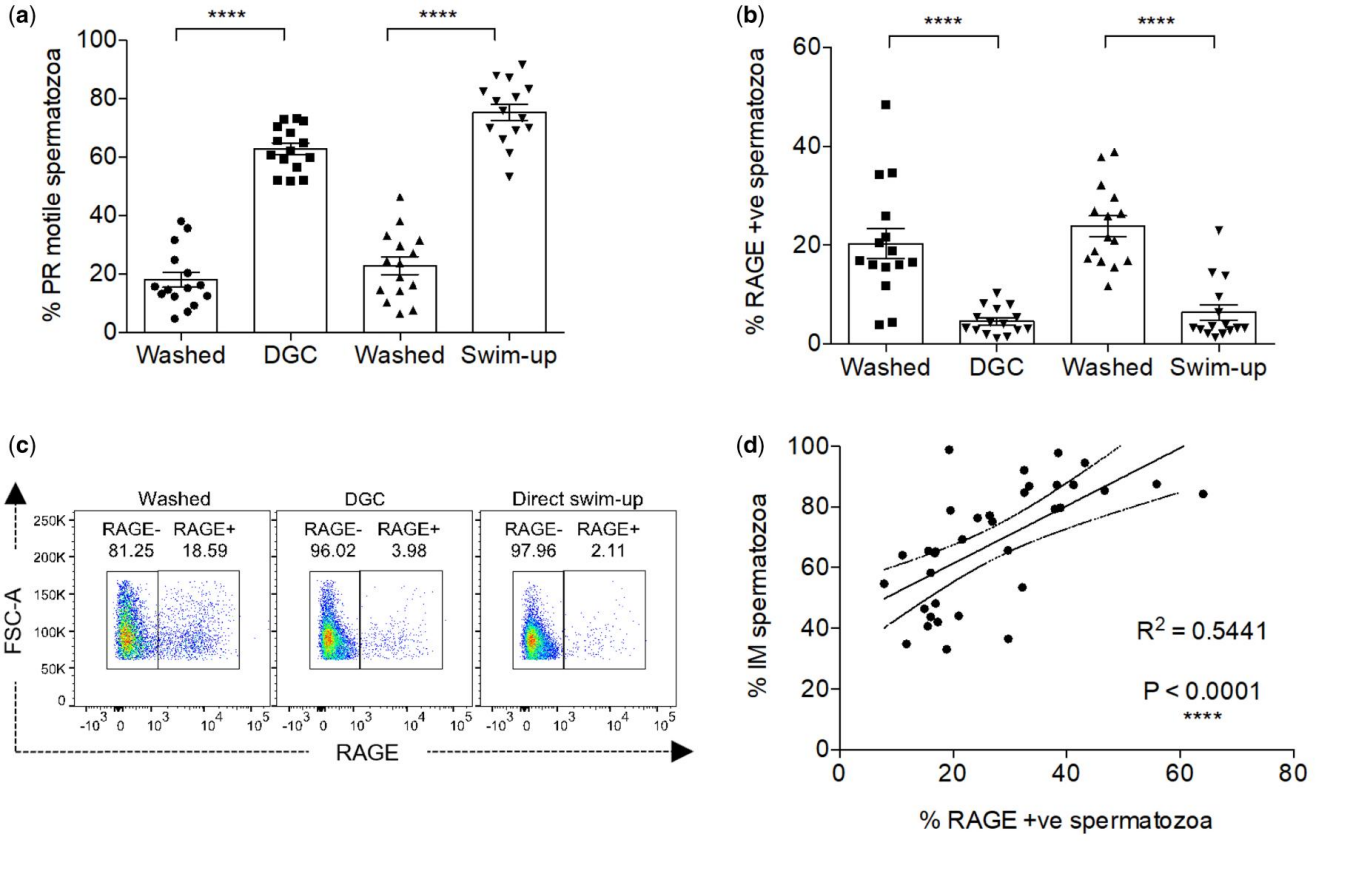
**RAGE expression in human spermatozoa is associated with the immotile sperm population**. (**a** and **b**) Proportion of (**a**) PR sperm and (**b**) sperm with RAGE expression in neat washed, direct swim-up and DGC purified semen samples, n = 15; % mean ± SEM. One-way ANOVA; ****P < 0.0001. (**c**) Representative flow cytometry data showing RAGE expression in neat washed, direct swim-up and DGC semen samples. (**d**) Correlation between total immotile sperm fraction and RAGE expressing sperm in washed semen samples, n = 35, error bars within 95% CI shown by dashed line. ****P < 0.0001. PR, progressively motile sperm; GC, gradient centrifugation; DGC, differential gradient centrifugation; PR, progressively motile sperm.

In accordance with WHO guidelines, we employed CASA to assess sperm motility in each semen sample and recorded the levels of immotile, non-progressive and progressive sperm. Progressively motile sperm represent the highest quality spermatozoa and can be enriched using one of two assays, DGC or the direct swim-up assay. Interestingly, we demonstrated that RAGE expression inversely associated with the levels of progressively motile sperm in purified semen samples (Fig. 2a–c). Specifically, the proportion of progressively motile spermatozoa increased from an average of 20.5 ± 10.9% in washed samples to 69.1 ± 11.9% (mean ± SD) in purified samples, for both purification methods (Fig. 2a).

In contrast, the percentage of RAGE expressing spermatozoa decreased from an average of 22.1 ± 10.1% in washed samples to 5.5 ± 4.8% (mean ± SD) in purified samples (Fig. 2b and c). This suggests that the low levels of RAGE in the purified samples are associated with sperm progressive motility and improved cell health. In a larger cohort of samples, it was also observed that RAGE expression correlates with poor sperm motility (Fig. 2d) which indicates that RAGE could be used to distinguish between normal and abnormal sperm at a cellular level.

**Figure 3.**
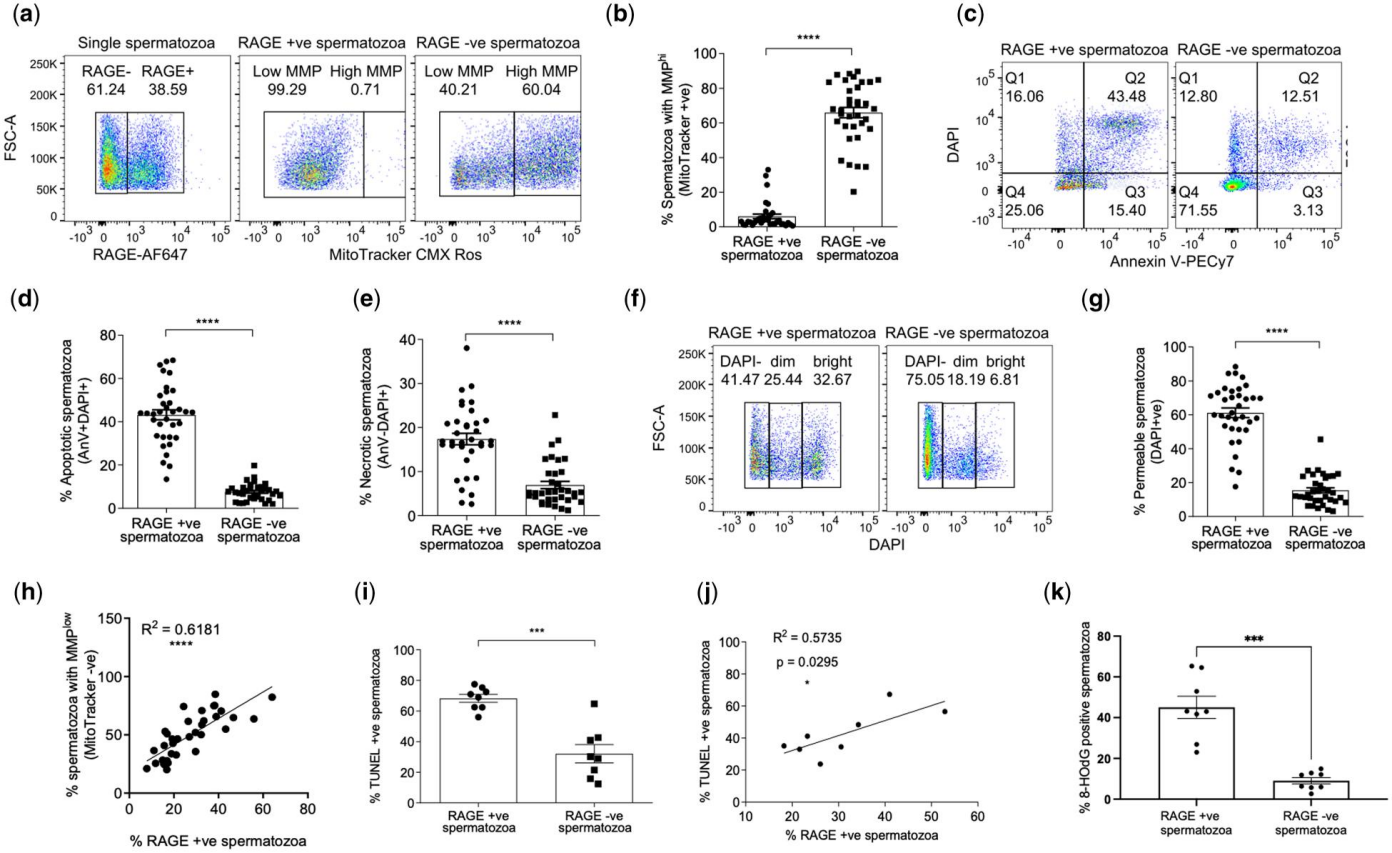
**RAGE expression in human spermatozoa is linked to multiple markers of poor sperm health**. Representative flow cytometry data and quantitative analysis of RAGE positive and negative populations relative to (**a** and **b**) MMP as measured by MitoTracker CMXRos, (**c**–**e**) apoptosis or necrosis as measured by AnnexinV–DAPI and (**f** and **g**) cell permeability as measured by DAPI fluorescence in washed samples, n = 35; % mean ± SEM. Paired *t*-test; ****P < 0.0001. (**h**) Correlation between low MMP and RAGE expression in washed sperm samples, n = 35, 95% CI shown by dashed line. ****P < 0.0001. (**i**) DNA fragmentation defined as a proportion of TUNEL positive spermatozoa in the RAGE positive and negative subgroups, n = 8; % mean ± SEM. Paired *t*-test; ***P < 0.001. (**j**) Correlation between TUNEL positive sperm and RAGE expressing sperm, n = 8, 95% CI shown by dashed line. *P < 0.05. (**k**) Oxidative DNA damage as determined by the number of 8-HOdG positive spermatozoa levels in RAGE positive and RAGE negative subgroups, n = 8, % mean ± SEM. Paired *t*-test, ***P < 0.001. MMP, mitochondrial membrane potential, 8-HOdG, 8-hydroxy-2-deoxyguanosine.

To provide a direct evaluation of RAGE expression against other markers of sperm health, analyses at a single cell level of cell permeability, mitochondrial function and cell death were conducted in parallel by flow cytometry (World Health Organization, 2021) (Supplementary Fig. S1). These analyses revealed that sperm mitochondrial function, cell death (via apoptosis and necrosis) and membrane integrity were greatly improved in the purified samples compared to washed samples (Supplementary Fig. S2). Earlier data showed an improvement in cell motility and a reduction in RAGE expression in purified sperm samples. In a larger cohort of samples, using flow cytometry, it was observed that RAGE expression on spermatozoa is associated with low-quality sperm as shown by a decrease in mitochondrial function (10-fold) and elevation in apoptotic- (6-fold) and necrotic-markers (2.5-fold) and a decrease in membrane integrity (3-fold) in the RAGE positive populations compared to RAGE negative populations (Fig. 3a–h).

Our data suggest that surface expression of RAGE is a biomarker of low-quality sperm. Similar to a previous study in diabetic men (Karimi *et al.*, 2011), it was also observed that the levels of DNA fragmentation, analysed using the TUNEL assay, were significantly higher (2.5-fold) in RAGE positive spermatozoa compared to RAGE negative spermatozoa (Fig. 3i) in non-diabetic men. In addition, the data shows that membrane-bound RAGE expression in human sperm correlates with DNA fragmentation measured by TUNEL (Fig. 3j) and is associated with oxidative damage to sperm DNA as measured by 8-HOdG (Fig. 3k) and as such, could be used as an indirect marker of DNA integrity and sperm cell health.

## Discussion

In this study, we show that in non-smoking, non-diseased males, the expression of RAGE on the sperm cell surface is linked to low-quality sperm. RAGE protein is a pivotal receptor in the body's inflammatory and oxidative stress responses. Upon activation, it initiates a signalling cascade that amplifies inflammatory signals, leukocyte recruitment and immune system activation through the production or a variety of pro-inflammatory cytokines, chemokines and cell surface proteins (Dong *et al.*, 2022). Simultaneously, RAGE activation increases the production of reactive oxygen species, contributing to oxidative stress and the generation of cellular damage, including lipid peroxidation, protein modification, and DNA fragmentation (Reddy, 2023).

Here, we affirm the localization of RAGE protein on the sperm cell surface (acrosomal and equatorial regions) and demonstrate a direct link between the expression of the membrane-bound form and low-quality sperm in non-obese, non-diabetic, non-smoking males. Our data show that RAGE expressing spermatozoa are effectively removed following selection of highly progressively motile spermatozoa in DGC and direct swim-up preparations. Further to this, through a series of flow cytometry studies, we could show that membrane-bound RAGE directly correlates with high-cell permeability, decreased mitochondrial function and higher levels of apoptosis and DNA fragmentation, at a cellular level.

Total RAGE expression on sperm has been previously studied in the context of diabetes (Mallidis *et al.*, 2007; Karimi *et al.*, 2011). This study is the first to focus specifically on the membrane-bound form of the RAGE protein in sperm in the absence of compounding factors such as obesity, smoking or diabetes, which are known to negatively affect sperm health (Fariello *et al.*, 2012; Palmer *et al.*, 2012; Dupont *et al.*, 2013). We could demonstrate in samples purified by DGC that the expression of membrane RAGE remained stable over time, indicating that the expression of RAGE on sperm is likely to be related to the health of the individual and its levels are set during spermatogenesis in the seminiferous tubules of the testicles. In this regard, factors such as diet and oxidative stress are known to induce glycated proteins and lipids that in turn can bind to RAGE receptors and promote not only inflammatory and oxidative stress responses but also establish a positive feedback loop that upregulates the expression of RAGE itself (Dong *et al.*, 2022). Therefore, the current work both extends and clarifies earlier findings and provides a highly specific approach to examining sperm health at a cellular level which could be relevant to quantifying fertility outcomes. The relationship between general health, RAGE protein expression, and sperm quality is a focus of our ongoing investigations.

In the recent decades, a sharp drop in sperm quality and sperm count has been observed globally, particularly in Europe, North America, Australia, and New Zealand, causing concerns about the negative impact of modern lifestyles and industrialization on male fertility (Nassan *et al.*, 2018; Salas-Huetos *et al.*, 2019; Fonseca *et al.*, 2022; Levine *et al.*, 2023). Evidence in humans shows that a high-fat diet and health conditions such as obesity, insulin resistance and diabetes are all co-incident with male infertility, with the major contributory factor being sperm DNA damage (Agbaje *et al.*, 2007; Fariello *et al.*, 2012; La Vignera *et al.*, 2012; Palmer *et al.*, 2012; Rama Raju *et al.*, 2012; Roessner *et al.*, 2012; Dupont *et al.*, 2013; Agarwal *et al.*, 2020; Levine *et al.*, 2023; De Jonge *et al.*, 2024). Moreover, exposure to lifestyle related toxins such as alcohol, tobacco, and cannabis or poor diet, reportedly plays an important role in development of male infertility, with studies suggesting that paternal environment and sperm epigenome may also have an impact on the health of his progeny (Lismer and Kimmins, 2023; De Jonge *et al.*, 2024). Strikingly, a number of recent studies declare that male reproductive health functions as a biomarker of future health, as infertile men seem to be more susceptible to certain diseases, such as cardiovascular conditions, genetic syndromes, and several cancers, which further accentuates the importance of extended research in this area (Eisenberg *et al.*, 2015a,b; Behboudi-Gandevani *et al.*, 2021; Hansen *et al.*, 2023; De Jonge *et al.*, 2024).

Advanced glycation end products (AGEs) are produced through non-enzymatic reactions between sugars and proteins, lipids or nucleic acids (Chen *et al.*, 2019; Prasad *et al.*, 2019; Darmishonnejad *et al.*, 2024). They are produced *in vivo* through a normal metabolic process but are also present in food, in particular in the modern carbohydrate- and saturated fat-rich diet, which may lead to an increased accumulation of AGEs in the body. This in turn impacts the metabolic status of an individual, which, as mentioned above, affects the sperm quality (Chen *et al.*, 2019; Prasad *et al.*, 2019; Ravichandran *et al.*, 2019; Kuzan, 2021; Darmishonnejad *et al.*, 2024). Interestingly, recent studies on rodents have confirmed the links between AGEs, RAGE, and low sperm quality. C57BL/6J mice fed an AGE-rich diet showed an increase in RAGE and carboxymethyl lysine (a glycated RAGE ligand), alongside increased body weight, elevated fasting glucose levels and adverse effects on sperm quality (Darmishonnejad *et al.*, 2024). In BALB/c mice, an AGE-rich diet induced histopathological damage in the testes and epididymis, alongside a decrease in total number of epididymal sperm, and an increase in abnormal sperm morphology and RAGE levels in the testes (Chen *et al.*, 2019). Meanwhile, Sprague-Dawley rats on an AGE-diet exhibited an increase in abnormal sperm morphology and a decrease in total epididymal sperm count, which was restored to control levels with Silymarin, an AGE inhibitor (Chen *et al.*, 2019). This further underlines the role AGEs and RAGE play in male infertility and the close links that exist between AGEs/RAGE, the overall metabolic health of the male, and their fertility status.

Current methods to identify unhealthy sperm include evaluating a range of physical parameters (morphology, motility, pH, viscosity, etc.), DNA fragmentation (TUNEL, COMET, AO, SCSA, SCD, etc.), sperm agglutination, sperm vitality, semen protein and leukocyte content among others. Evaluation of RAGE expression could be used in conjunction with these approaches or used independently in a clinical setting to provide a simple means to detect male infertility or it could be used as a molecular marker to purify high-quality sperm for assisted reproduction.

## Supplementary Material

hoae064_Supplementary_Data

## Data Availability

The data underlying this article will be shared on reasonable request to the corresponding author.
