# Supplementary material for: Membrane-bound receptor for advanced glycation end products (RAGE) is a stable biomarker of low-quality sperm
Source: Hum Reprod Open. 2024 Nov 7;2024(4):hoae064. doi: 10.1093/hropen/hoae064 (PMC11568349; doi:10.1093/hropen/hoae064)
Supplement: hoae064_Supplementary_Data [file hoae064_supplementary_data.pdf]

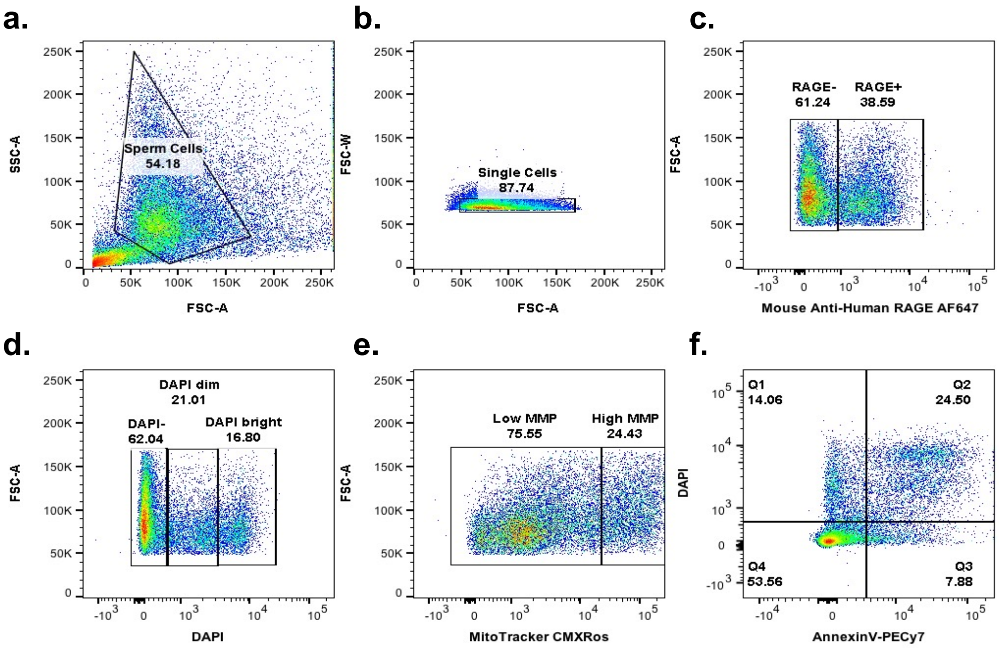

Supplementary Figure S1. Gating strategy for flow cytometry assays.

(a) Sperm cells were gated in the flame shaped region using FSC-A *vs* SSC-A parameters. **(b)** Single cells were gated using FSC-A *vs* FSC-W parameters. **(c)** RAGE negative and positive cells were selected using rectangle gates. **(d)** DAPI negative, dim and bright cells were selected using rectangle gates. **(e)** High and low MitoTracker CMXRos were selected using rectangle gates. **(f)** Apoptotic cells were gated using Annexin V/DAPI staining separated into four quadrants. FSC-A - forward scatter area , SSC-A – side scatter area, FSC-W – forward scatter width.

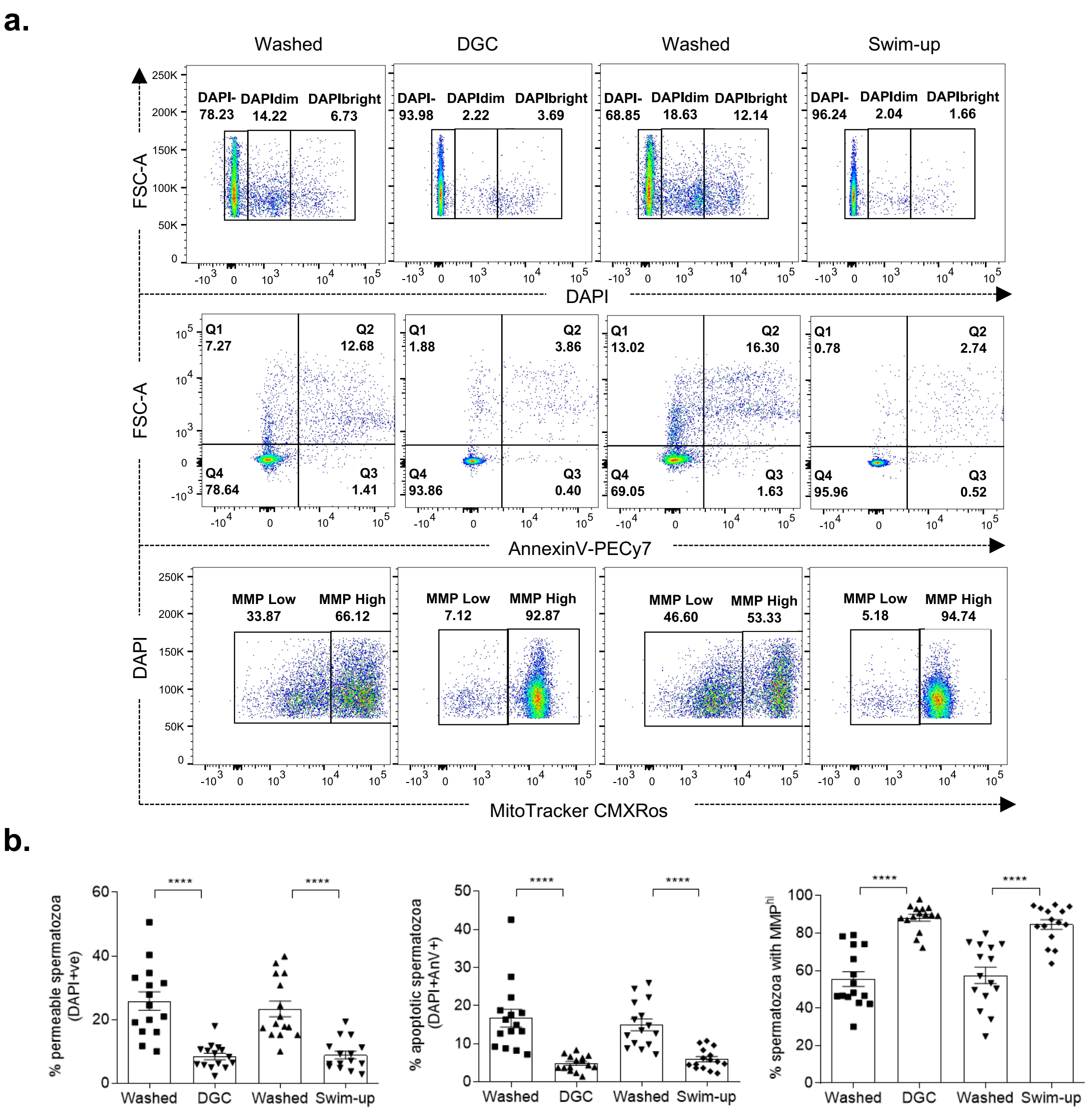

Supplementary Figure S2. Quantification of sperm cell health by flow cytometry in washed and purified spermatozoa.

(a) Representative flow cytometry data showing DAPI, MitoTracker CMXRos, and AnnexinV fluorescence in different spermatozoa preparations: washed, DGC purified and purified through direct swim-up. **(b)** Cell permeability, apoptosis and mitochondrial membrane potential measurement in washed, DGC and direct swim-up purified sperm, n = 15; % mean ± SEM. One-way ANOVA; ****p<0.0001. DGC – differential gradient centrifugation.
